# Buffalo long non-coding RNA gene11007 promotes myoblasts proliferation

**DOI:** 10.3389/fvets.2022.857044

**Published:** 2022-08-05

**Authors:** Ning Zhang, Gaoxiao Xu, Ping Sun, Shuzhe Wang, Yunchang Zhu, Saixing Duan, Mingsheng Jiang, Hui Li, Xuefeng Wei, Yun Ma

**Affiliations:** ^1^Ningxia Key Laboratory of Ruminant Molecular and Cellular Breeding, School of Agriculture, Ningxia University, Yinchuan, China; ^2^State Key Laboratory of Agricultural Microbiology, Huazhong Agricultural University, Wuhan, China; ^3^Anhui Province Key Laboratory of Embryo Development and Reproductive Regulation, Anhui Province Key Laboratory of Environmental Hormone and Reproduction, School of Biological and Food Engineering, Fuyang Normal University, Fuyang, China; ^4^State Key Laboratory for Conservation and Utilization of Subtropical Agro-Bioresources, College of Animal Science and Technology, Guangxi University, Nanning, China; ^5^College of Life Sciences, Xinyang Normal University, Institute for Conservation and Utilization of Agro-Bioresources in Dabie Mountains, Xinyang, China

**Keywords:** buffalo, RNA-Seq, lncRNA, myogenesis, proliferation

## Abstract

Buffalo meat is of good quality because it is lean and tender, and could bring significant cardiovascular benefits. The underlying difference in muscle development and meat quality is a complex and precisely orchestrated process which has been demonstrated to be regulated by long non-coding RNAs (lncRNAs). However, the regulatory role of lncRNAs in the growth and development of buffalo skeletal muscle is still unclear. In this study, the Ribo-Zero RNA-Seq method was used to explore the lncRNA expression profiles of buffalo myoblasts during the proliferation and differentiation phases. A specific set of 9,978 lncRNAs was found. By comparing the expression profiles of lncRNAs, it was found that there were 1,576 differentially expressed lncRNAs (DELs) during buffalo myoblast differentiation. Twelve DELs were chosen and subsequently verified in eight different buffalo tissues during fetal and adult stages by using qPCR. Gene11007 was found to be one of the most down-regulated lncRNAs during buffalo myoblasts differentiation and it was subsequently characterized. EdU, CCK-8, qPCR and western blotting assays showed that gene11007 promoted the proliferation of buffalo myoblasts but it had no effect on cell differentiation. Our research may enrich the genome annotations of buffalo and provide a new molecular target for the in-depth understanding of the regulation of lncRNAs in skeletal muscle.

## Introduction

The possibility to improve the quality and tenderness of meat products is of major importance to the meat industry. The fundamental solution to this problem probably lies at the molecular level during the growth phase of the muscle which ultimately determines the quality of beef produced. As the problem of tenderness variability, improving the meat quality is of major importance in the meat industry (1). From the perspective of the molecular mechanism of meat, how to improve the quality of beef is the most fundamental solution (2). Meat quality trait is governed by protein-coding and non-coding genes that results in muscle development and fat deposition (3, 4). High-throughput sequencing provides an approach to studying differences in genes function (5, 6). Two typical Chinese indigenous bovine species including cattle (*Bos taurus*) and buffalo (*Bubalus bubalus*) have great potential for meat production and are worthy of development. China's buffalo stock is 27.44 million, making it the third largest buffalo breeding country in the world. With the rapid improvement of agricultural mechanization, the labor value of buffalo is gradually decreasing. Therefore, the possibility of using this resource for meat production and further increase beef production has become one of the important tasks of current buffalo breeding (7). The investigation of the molecular mechanisms involved in the regulation of quality traits of meat in order to improve China's beef cattle breeding program has become a major priority.

Skeletal muscle is an important part of livestock and poultry meat, and its muscle fiber type composition is a vital factor that contributes to the meat quality for meat quality (8, 9). Therefore, studying skeletal muscle development has certain guiding significance for how to improve meat production and quality. At the embryonic stage, Pax3+/Pax7+ myogenic progenitor cells migrate from the mesoderm (10, 11). The myogenic regulatory factors Myf5, MyoD and MRF4 are involved in the regulation of myogenic determination. The myogenic cells further proliferate and differentiate to form muscle fibers. After birth, the number of muscle fibers has already been determined, and the increase in muscle mass depends mainly on their growth and thickening (12, 13). When skeletal muscles are injured, they have the ability to regenerate and repair, and this depends mainly on the activation of the myogenic stem cells (satellite cells, Pax7+/Myf5-). The activated satellite cells can differentiate into myoblasts and fuse to form new muscle fibers for the purpose of repairing injured muscle fibers. Whether the skeletal muscle formation occurs at the embryonic stage or there is regeneration of skeletal muscle after birth, apart from the regulation of Pax3/Pax7 and MRFs, these processes are also affected by the MEF2 family, Wnt, IGFs and non-coding RNAs (lncRNAs) (14, 15).

Long ncRNAs (lncRNAs) are > 200 nt in length and are a type of RNA which has minimal or no protein coding ability (16–18). They can regulate gene expression and activity at the transcription and post-transcriptional levels. Many studies have reported that lncRNAs are involved in regulating the growth and development of skeletal muscle (19–21). LncRNAs usually exhibit different regulatory mechanisms according to their cellular location (22). In the nucleus, lncRNAs can be used as a molecular scaffolds and decoys in order to bind regulatory elements or recruit transcription factors to mediate gene transcription. For example, the lncRNA, Dum, recruits DNA methyltransferase to the promoter of developmental pluripotency-associated 2 (Dppa2), thereby inhibiting the transcriptional activity of the *Dppa2* gene (23). In the cytoplasm, lncRNAs can be used as competitive endogenous RNAs to bind microRNAs (miRNAs), thereby increasing the expression of target genes (24–26). For example, lncRNA MDNCR acts as a molecular sponge to competitively bind miR-133a, thereby inducing the differentiation of bovine myoblasts (17, 26). Although a large number of functional lncRNAs involved in regulation of myogenesis have been identified in humans and mice, there is little known regarding lncRNAs in buffaloes (27–29).

The proliferation and differentiation of buffalo myoblasts are the molecular basis for the increase in muscle mass. This study aimed to obtain lncRNAs that are differentially expressed during the proliferation and differentiation phases of buffalo myoblasts by using RNA-seq technology. Several thousand lncRNAs were identified and many of these were found to be specifically expressed in buffalo myoblasts and muscle tissues. We characterized the functions of one down-regulated lncRNA, gene11007, which induced buffalo myoblast proliferation. This study not only provides genetic resources for in-depth understanding of the regulatory effects of lncRNAs on muscles, but also provides a new perspective for screening and obtaining molecular targets for buffalo breeding.

## Materials and methods

### Sample preparation

Embryo and adult buffalo tissues (heart, liver, brain, spleen, lung, kidney, leg muscle, and musculus longissimus) were collected and rapidly frozen in liquid nitrogen. The research protocol and animal care for this study was approved by the Animal Care Committee of the College of Agriculture of Ningxia University.

### Preparation of libraries and illumina sequencing

RNA was extracted from three samples of buffalo myoblasts each arrested during the proliferation and differentiation phases of growth. Proliferation were grown to 50% confluence in growth medium and differentiation myoblasts were induced to differentiate for 4 days in differentiation medium. The RNA concentrations in the samples were quantified using an Agilent 2100 Bioanalyzer and NanoDrop spectrophotometer. A Ribo-Zero™ rRNA Removal Kit (Epicenter, Madison, WI, USA) was used to remove ribosomal RNA. Library preparation and sequencing analysis were performed as described previously. The raw data files were cleaned up for quality control through Trim Galore and then mapped to the buffalo reference genome (UOA_WB_1 assembly, https://www.ncbi.nlm.nih.gov/genome). CuffLinks were used for linear transcripts assembly and abundance assessment, and then the reads from fusion transcripts that did not match the linear RNA sequence were identified. The lncRNAs ≤ 100 kb in length, with at least two support sequences, and sequences with no more than two mismatches were retained for further analysis.

### Gene ontology and pathway analysis

Gene Ontology (GO) analysis (http://www.geneontology.org) was used to explore potential functions of differentially expressed mRNAs and lncRNAs. In addition, Kyoto Encyclopedia of Genes and Genomes (KEGG) pathway analysis (http://www.kegg.jp) was also performed, and DAVID was used to gain insight into the interactions and reaction networks of differentially expressed lncRNA molecules. The –log_10_*P* value represents a significant enrichment of a given GO term or KEGG pathway between up-regulated and down-regulated entities.

### CDNA synthesis and real-time quantitative PCR

The total RNA was extracted from buffalo tissues and myoblasts using Trizol reagent (TaKaRa, Dalian, China). PrimeScript™ RT kit (TaKaRa, Dalian, China) with gDNA eraser was used to reverse transcribe the total RNA while removing genomic DNA. The SYBR Green kit (TaKaRa, Dalian, China) was used to perform qPCR in triplicate on a Bio-Rad CF96 system (Bio-Rad, USA), and the data was normalized using β*-actin*. The primers used are listed in Supplementary Table S1.

### Plasmid construction

The full length of gene11007 (Supplementary Text S1) was ligated intothe pcDNA3.1(+) vector in order to construct an over-expression plasmid (Supplementary Table S1). The recombinant plasmid was confirmed by sequencing analysis (Sangon Biotech, Shanghai, China).

### Cell culture and treatments

Isolation and culture of buffalo myoblasts were carried out according to existing methods (30–33). The procedure used was as follows: the longissimus dorsi muscle tissues of the 3 months old buffalo fetuses were collected and cut into fragments after the fascia fat was removed. The muscle tissue was washed with PBS and digested with 300 U of collagenase I (Gibco, Waltham, MA, USA, Waltham, MA, USA) in a water bath at 37 °C for about 1 h. After digestion, the mixture was centrifuged at 600 *g* for 5 min, the supernatant was discarded and 0.25% trypsin of equal volume was added for further digestion at 37 °C for 20 min. After this step a viscous liquid formed and DMEM medium (Gibco, Waltham, MA, USA) containing 10% FBS (Gibco, Waltham, MA, USA) was added to terminate the digestion, and the mixture was filtered successively through 100, 70 and 40 mesh filters, respectively. After a further centrifugation at 600 *g* for 5 min, the supernatant was discarded and washed 2-3 times with PBS. After another centrifugation, the cells were re-suspended with medium containing 20% fetal bovine serum and 1% penicillin and streptomycin. Finally, the cells were inoculated into cell culture dishes and cultured in an incubator at 37 °C with 5 % CO_2_ for 2 h. After that, the supernatant was transferred to a new dish to obtain purified myoblasts. Myoblast differentiation was induced with 2% horse serum medium (Gibco, Waltham, MA, USA), and the buffalo myoblasts were replaced with fresh medium every day, and the cells were induced for 4 days for cell differentiation experiments.

### Fluorescence in situ hybridization

FISH (ServiceBio Co. Ltd., Wuhan, China) was conducted as follows: buffalo myoblast cells were cultured to 70 % confluence and then treated with *in situ* hybridization fixation solution for 30 min as instructed. The prepared premix was incubated with the cells at 37 °C for 1 h. The fixed cells were then incubated overnight with a mixed solution containing the gene11007 probe. After the cells were washed 2-3 times with PBS, DAPI solution was added to the cells in a darkened environment for 8 min. Finally, the cells were observed using a fluorescent microscope (Nikon, Tokyo, Japan).

### EdU and CCK-8 assays

A CCK-8 (Vazyme, Nanjing, China) and the Cell-Light™ Eduapollo ®567 *in vitro* imaging kit (RiboBio, Guangzhou, China) were used to study the proliferation status of buffalo myoblasts. The detailed procedures used strictly followed the manufacturer's instructions. EdU (n = 5) and CCK-8 (n = 5) assays were performed at 18 h after transfection.

### Western blotting analysis

Protein was extracted using RIPA buffer containing 1 % PMSF (SolarBio, Beijing, China). A BCA kit (Beyotime, Shanghai, China) was used to determine the protein concentration. The isolated proteins were transferred to polyvinylidene fluoride membranes after 10 % SDS polyacrylamide gel electrophoresis. The membranes were incubated overnight with primary antibodies to cyclin D1 (Wanlei, Shenyang, China), PCNA (Wanlei, Shenyang, China), CDK2 (Abcam Ltd., Cambridge, MA, USA), MyoD (Abcam Ltd., Cambridge, MA, USA), myogenin (MyoG; Abcam Ltd., Cambridge, MA, USA), MyHC (Genetex, Southern California, USA) and β-actin (Abcam, Cambridge, UK) at 4 °C. The membranes were then incubated with the corresponding secondary antibodies at room temperature for 1 h. After washing, the membranes were exposed to ECL Plus (SolarBio, Beijing, China) and the Chemidoc XRS+ system (Bio-Rad, California, USA) was used for imaging the resultant bands.

### Statistical analyses

The data were expressed as the means ± SEMs. *P*-values were calculated byusing the Student's *t*-test and one-way ANOVA. *P* < 0.05 was considered significantly different.

## Results

### Expression profiles of LncRNAs in proliferating and differentiated buffalo myoblasts

**Figure 1:**
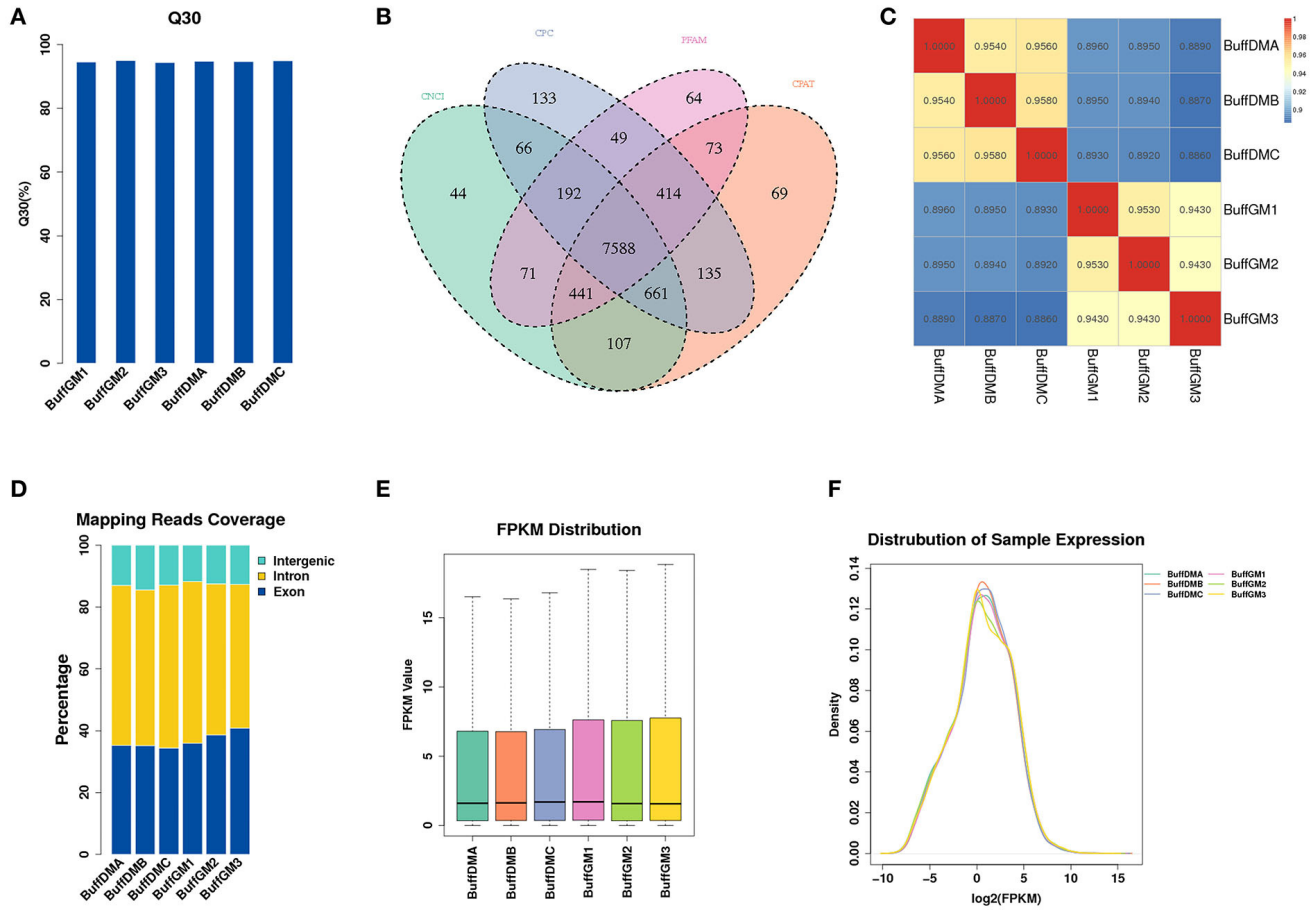
Identification of lncRNAs in proliferating and differentiating buffalo myoblasts. **(A)** The clean Q30 base rate (%). **(B)** A Venn diagram depicting the overlap of lncRNAs found in the six samples. **(C)** The performance of Ribo-Zero RNA-seq was evaluated by determining the correlation of lncRNA expression. **(D)** The classification of lncRNAs, as defined by their genomic localization. **(E,F)** The expression of lncRNAs in the six samples. BuffGM represents buffalo muscle cells at the proliferative phase and BuffDM represents buffalo muscle cells at the differentiation phase.

**Figure 2:**
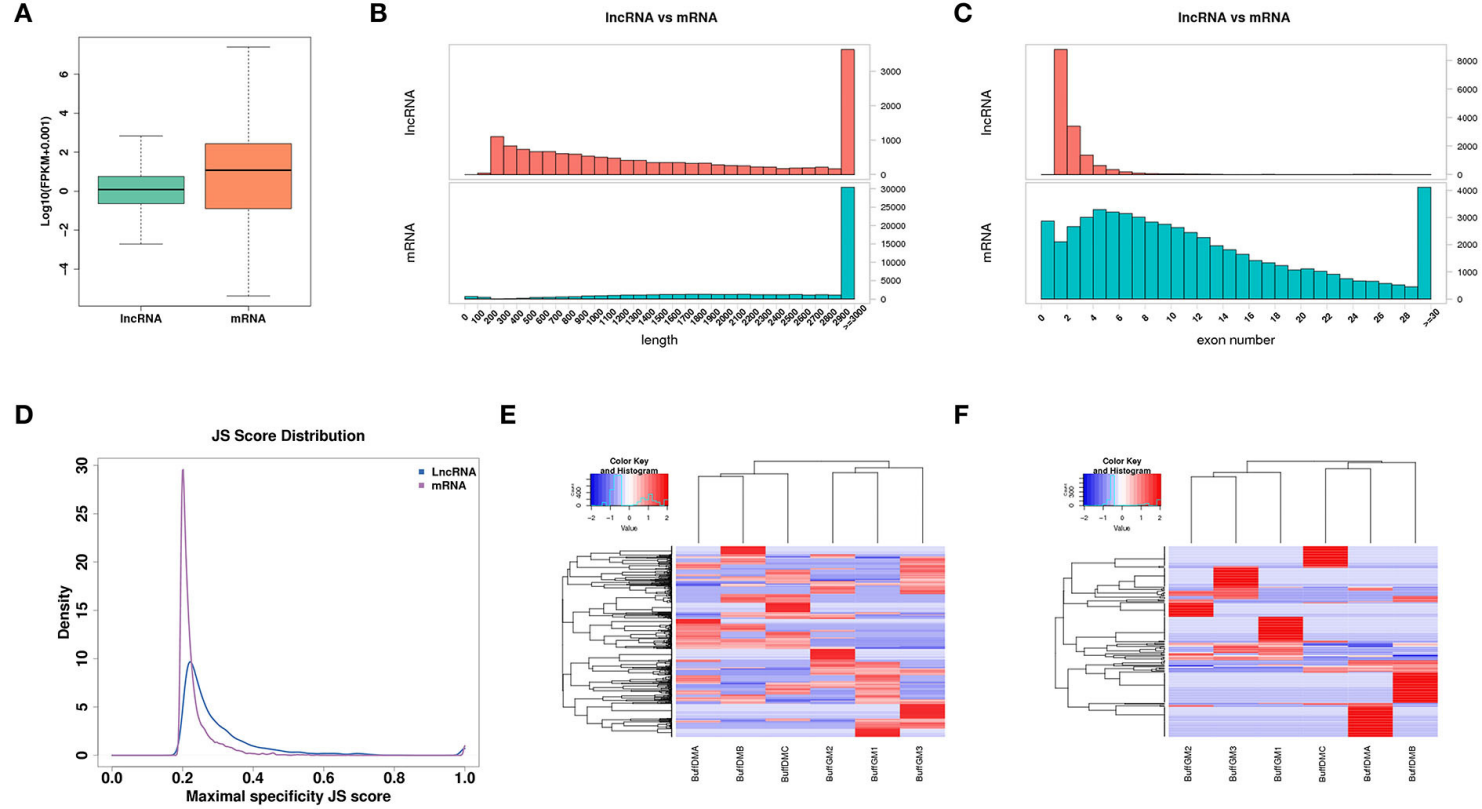
Expression profiles of lncRNAs in proliferating and differentiating buffalo myoblasts. **(A)** The distribution of expression levels of lncRNAs and mRNAs. **(B)** The distribution of transcript lengths of lncRNAs and mRNAs. **(C)** The distribution of exon number of lncRNAs and mRNAs. **(D)** A Jensen-Shannon divergence score was used to analyze the tissue expression specificity of lncRNAs and mRNAs. **(E,F)** The lncRNA and mRNA with JS scores > 0.5 were screened and a heatmap was drawn according to their expression levels to verify their expression trends and tissue expression specificities. BuffGM represents buffalo muscle cells at the proliferative phase and BuffDM represents buffalo muscle cells at the differentiation phase.

A large number of RNAs were identified with high accuracy in the proliferation and differentiation phases of buffalo myoblasts by using Ribo-Zero RNA-Seq (Q30 = 94.7%, Figure 1A). We used Pfam and Cpat databases to filter and analyzed the protein coding ability predictions of new lncRNAs through CPC and CNCI tools, and identified 9,978 potential lncRNA transcripts (Figure 1B, Supplementary Table S2). As shown in Figure 1C, Pearson correlation analysis showed that there is a good correlation between proliferating and differentiated buffalo myoblasts. 96.2~100 and 92.2~97.9 million mapped clean reads were obtained from the proliferating and differentiated buffalo myoblasts (Table 1), respectively. We found that 36.72% of reads were located in the exon regions, while the non-coding portions, including the intergene (12.87%) and intron regions (50.41%), dominated the distribution (Figure 1D). As shown in Figures 1E,F, the average expression level of lncRNA identified by RNA-seq was relatively low at 2.09 (FPKM; Supplementary Table S2). However, the protein-coding genes showed higher levels of expression (16.79; Figure 2A, Supplementary Table S3), suggesting that not every lncRNA expressed in muscle plays an important regulatory role, but this does not affect the role of lncRNA as an important player in skeletal muscle development.

**Table 1:** Summary of mapping the reads to the reference genome.

**Library**	**BuffDMA**	**BuffDMB**	**BuffDMC**	**BuffGM1**	**BuffGM2**	**BuffGM3**
Total reads	101,952,364	96,266,686	98,333,562	100,061,338	103,546,858	102,399,234
Mapped reads	97,884,766	92,213,475	94,448,761	96,226,375	100,023,977	98,508,495
Mapping rate	0.9601	0.9579	0.9605	0.9617	0.966	0.962
UnMapped reads	4,067,598	4,053,211	3,884,801	3,834,963	3,522,881	3,890,739
MultiMap reads	1,786,567	1,941,195	1,854,483	1,944,564	2,265,651	2,385,732
MultiMap rate	0.0175	0.0202	0.0189	0.0194	0.0219	0.0233

BuffGM and BuffDM represent buffalo muscle cells in the proliferative and differentiation stages, respectively.

As one of the types of the non-coding RNAs, lncRNAs tend to be shorter than the transcripts that can encode the protein. As shown in Figure 2B, the average length of lncRNA is shorter than that of mRNA. Similarly, compared with the protein-coding genes, lncRNAs have fewer exons (Figure 2C). In addition, we used the Jensen-Shannon divergence (JS) score to analyze the specificity of the corresponding tissue expression of the lncRNAs and protein coding genes, and found that they were similar (Figure 2D). Moreover, according to the JS scores of lncRNAs and mRNAs, the RNAs with JS scores >0.5 were screened and a heat map was drawn according to their expression levels in order to check their expression trends and tissue expression specificities (Figures 2E,F).

### Differentially expressed LncRNAs analysis

**Figure 3:**
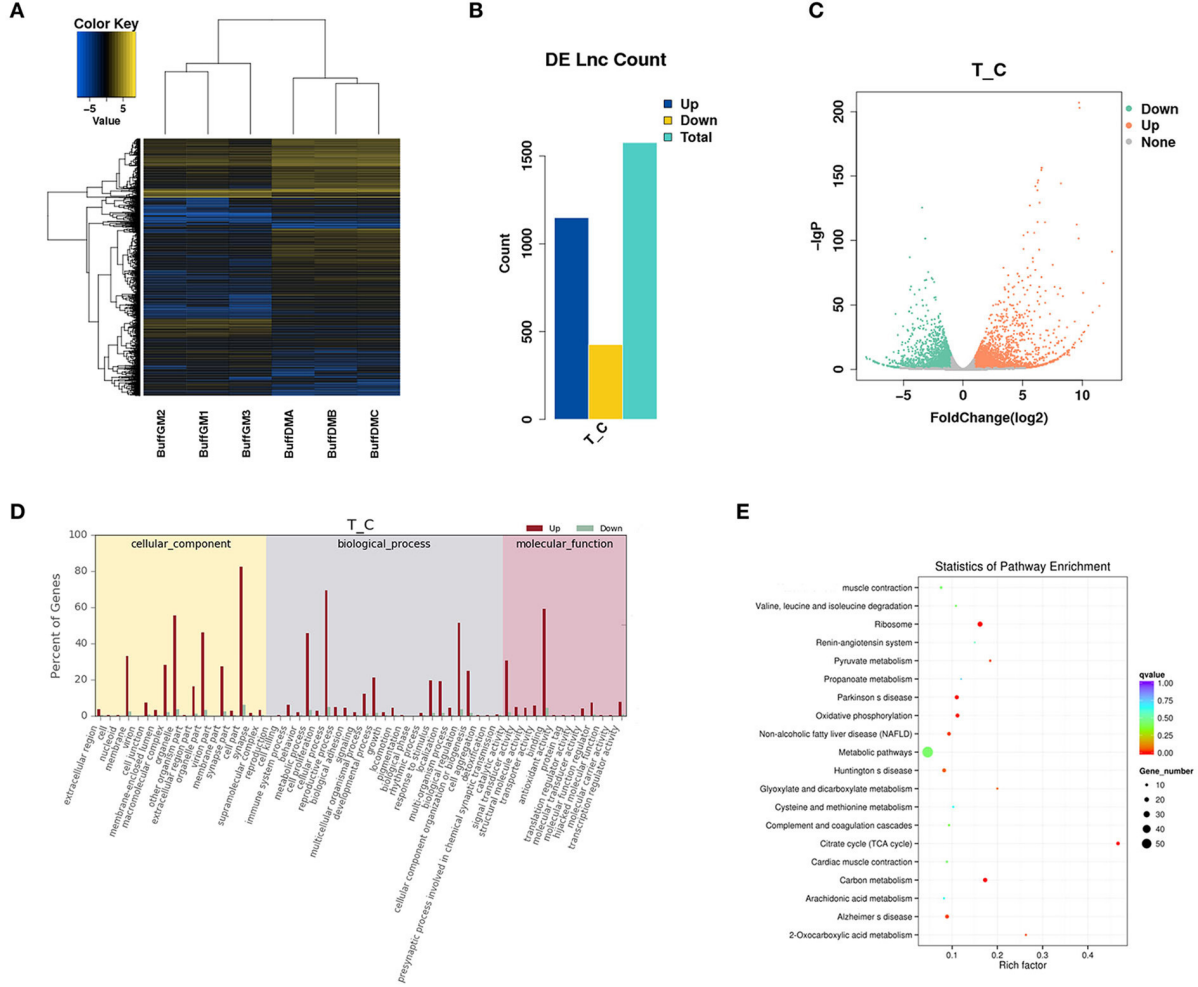
Differentially expressed lncRNAs in proliferating and differentiating buffalo myoblasts. **(A–C)** A heatmap, histogram and volcano plots of thedifferentially expressed lncRNAs in proliferating and differentiating buffalo myoblasts. **(D,E)** The enriched GO terms and KEGG pathways are shown. BuffGM represents buffalo muscle cells at the proliferative phase and BuffDM represents buffalo muscle cells at the differentiation phase.

In this study, a total of 1,576 differentially expressed lncRNAs (DELs) were screened (*P* < 0.05; Figure 3A, Supplementary Table S2). We found that 427 lncRNAs were down-regulated and 1,149 lncRNAs were up-regulated (Figure 3B). It is worth mentioning that MSTRG.118857 (FPKM = 3,518.98) and gene11007 (FPKM = 5,034.96) had the highest expression levels among all up-regulated and down-regulated lncRNAs, respectively. A volcano plot was drawn to better understand the expression changes and potential functions of lncRNAs (Figure 3C).

LncRNAs can affect the transcription and translation of coding genes in both *cis* and *trans* regulation. For example, when a lncRNA acts as a molecular scaffold on a chromosome, it can also perform *cis* regulation. However, when it affects gene expression on another chromosome, *trans* regulation can then allow it to play a role so as to act as a miRNA sponge. The enriched GO terms and KEGG pathways are shown in Figures 3D,E. Interestingly, many muscle development-related genes (*cis* and *trans*' target genes of DELs) were enriched by GO and KEGG analysis, indicating that lncRNAs are not simply acting as transcriptional noise, but have corresponding transcriptional or post-transcriptional regulatory effects.

**Figure 4:**
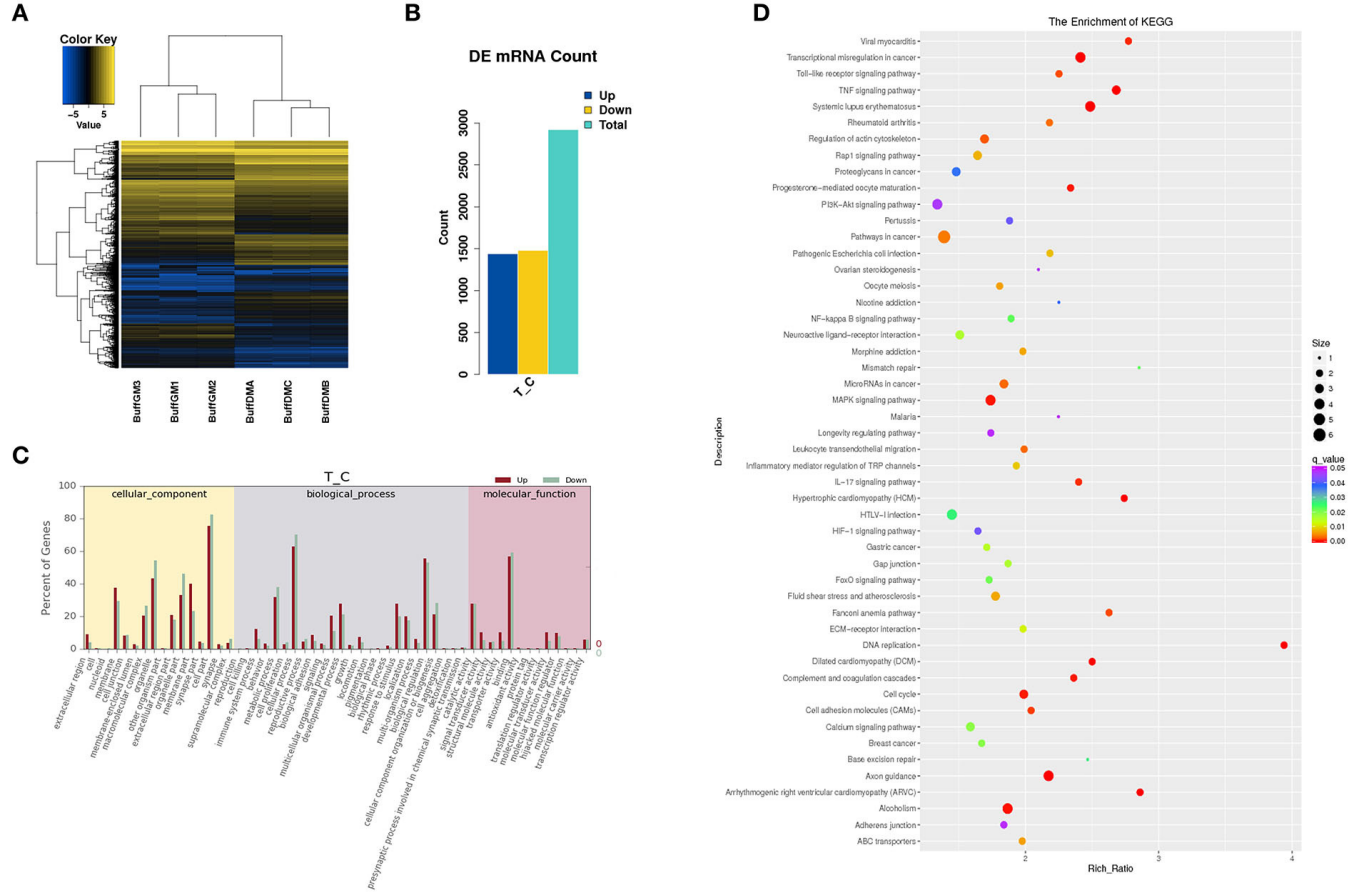
Differentially expressed mRNAs in proliferating and differentiating buffalo myoblasts. **(A,B)** A heatmap and histogram of the differentially expressed lncRNAs in proliferating and differentiating buffalo myoblasts. **(C,D)** The enriched GO terms and KEGG pathways are shown. BuffGM represents buffalo muscle cells at the proliferative phase and BuffDM represents buffalo muscle cells at the differentiation phase.

To further understand the potential role of differentially expressed protein-coding genes, we also analyzed the expression abundance of transcripts in buffalo myoblasts during the proliferation and differentiation phases (Figure 4). We found that 2,921 mRNAs were significantly different in expression (*P* < 0.05), and all the differentially expressed genes (DEGs) are shown in Supplementary Table S3. 1,440 mRNAs were up-regulated in samples from cells during the differentiation phase when compared to the proliferation stage, while 1,481 mRNAs were down regulated (Figures 4A,B). We performed GO and KEGG enrichment analysis to shed additional light on the potential functions of these DEGs. These GO annotations and pathways may be involved in the growth and development of buffalo skeletal muscles (Figures 4C,D).

### Identification of candidate LncRNAs

**Figure 5:**
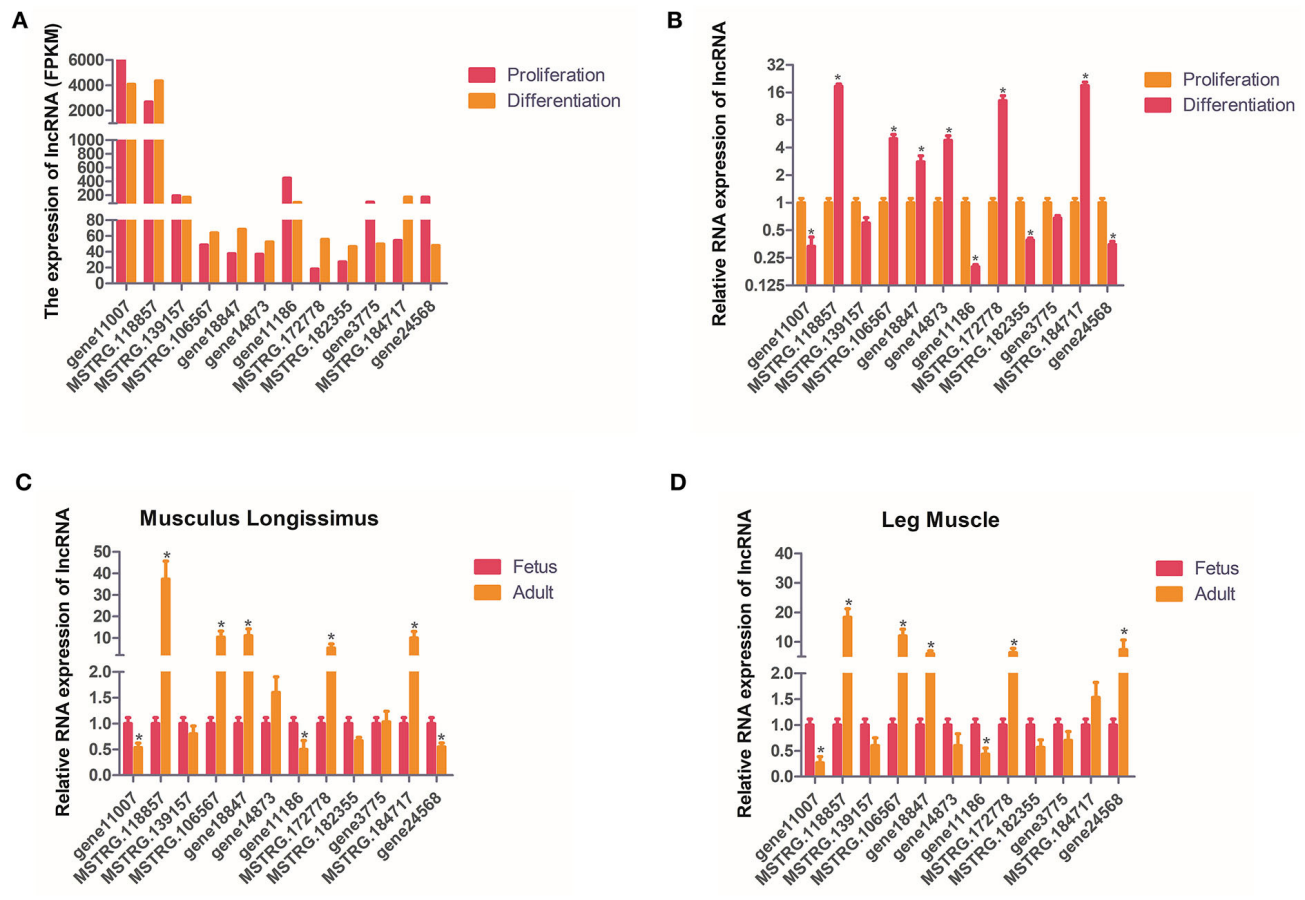
Validation of putative lncRNAs. **(A,B)** 12 candidate lncRNAs were selected and identified using quantitative real-time PCR. **(C,D)** 12 candidate lncRNAs were identified in fetal and adult buffalo leg muscles and musculus longissimus using qPCR. The values are means ± SEMs for three individuals. *represents *P* < 0.05.

In order to verify the accuracy of sequencing data, we randomly selected 12 DELs for qPCR and compared the data obtained with the sequencing data. It can be seen that the qPCR results of these lncRNAs are basically similar to the RNA-seq results, indicating the accuracy of the RNA-seq data (Figures 5A,B). The expression of these 12 lncRNAs were analyzed in the leg muscles and musculus longissimus during the fetal and adult stages, and it was found that the change in the trend of the lncRNAs in leg muscle was basically consistent with that in e musculus longissimus (Figures 5C,D). MSTRG.118857 and gene11007 had the highest expression levels among all up-regulated and down-regulated lncRNAs, respectively, indicating that these two genes may be important in buffalo muscle growth and development. The expression level of gene11007 in the proliferation phase of buffalo myoblasts was significantly higher than that in the differentiation phase, whereas MSTRG.118857 showed the opposite (Figures 5A,B). Gene11007 expressed higher in fetal bovine was than that of adult buffalo in both the longissimus dorsi muscle and leg muscle (Figures 5C,D). Quite the contrary, MSTRG.118857 is more abundant in adult buffalo. Therefore, we measured the expression of these 12 lncRNAs in different tissues of buffalo, in order to find potential lncRNAs specifically expressed in skeletal muscle.

**Figure 6:**
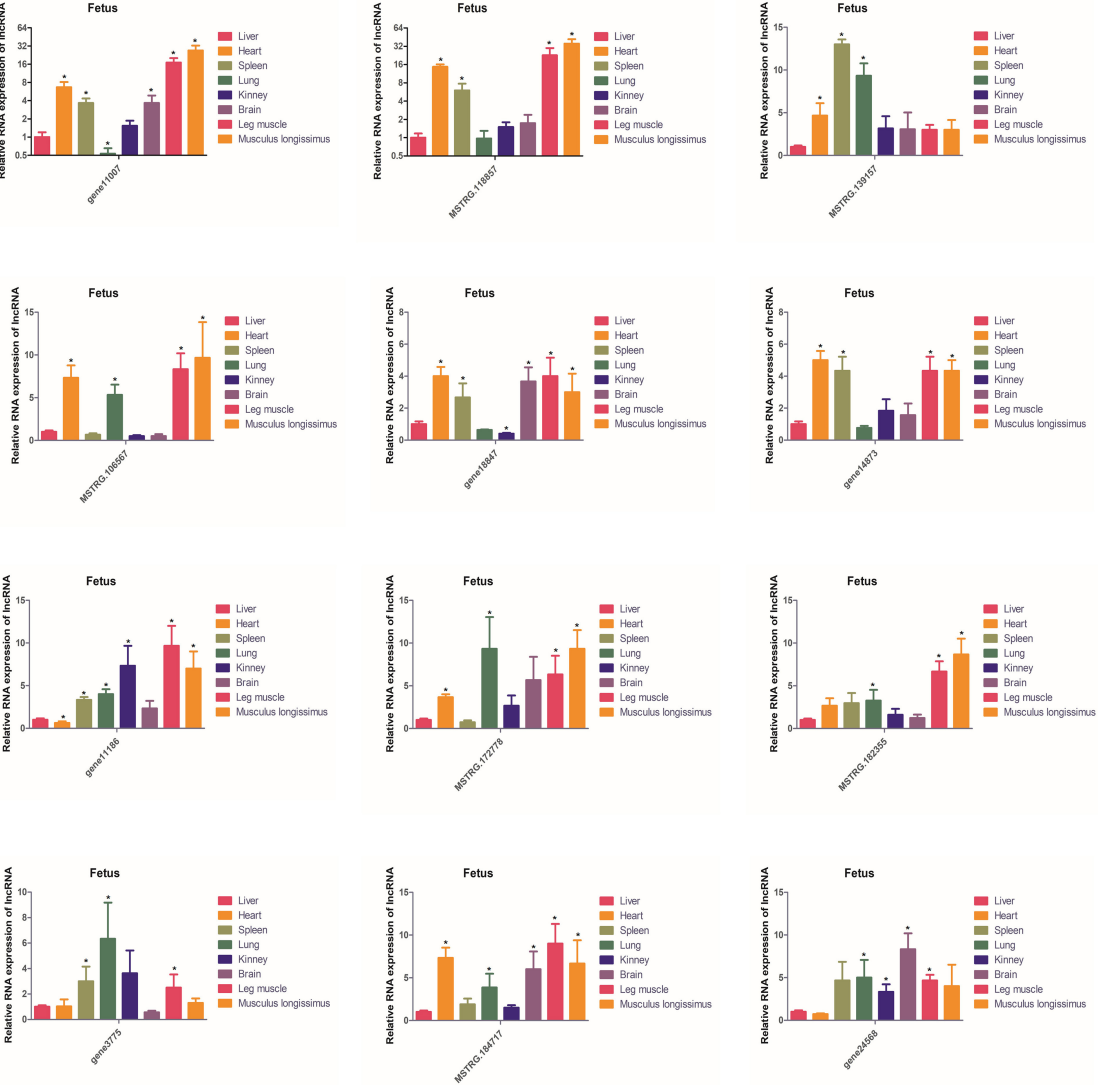
Expression levels of 12 candidate lncRNAs in different tissues obtained from fetal buffaloes. The values are means ± SEMs for three individuals. *represents *P* < 0.05.

**Figure 7:**
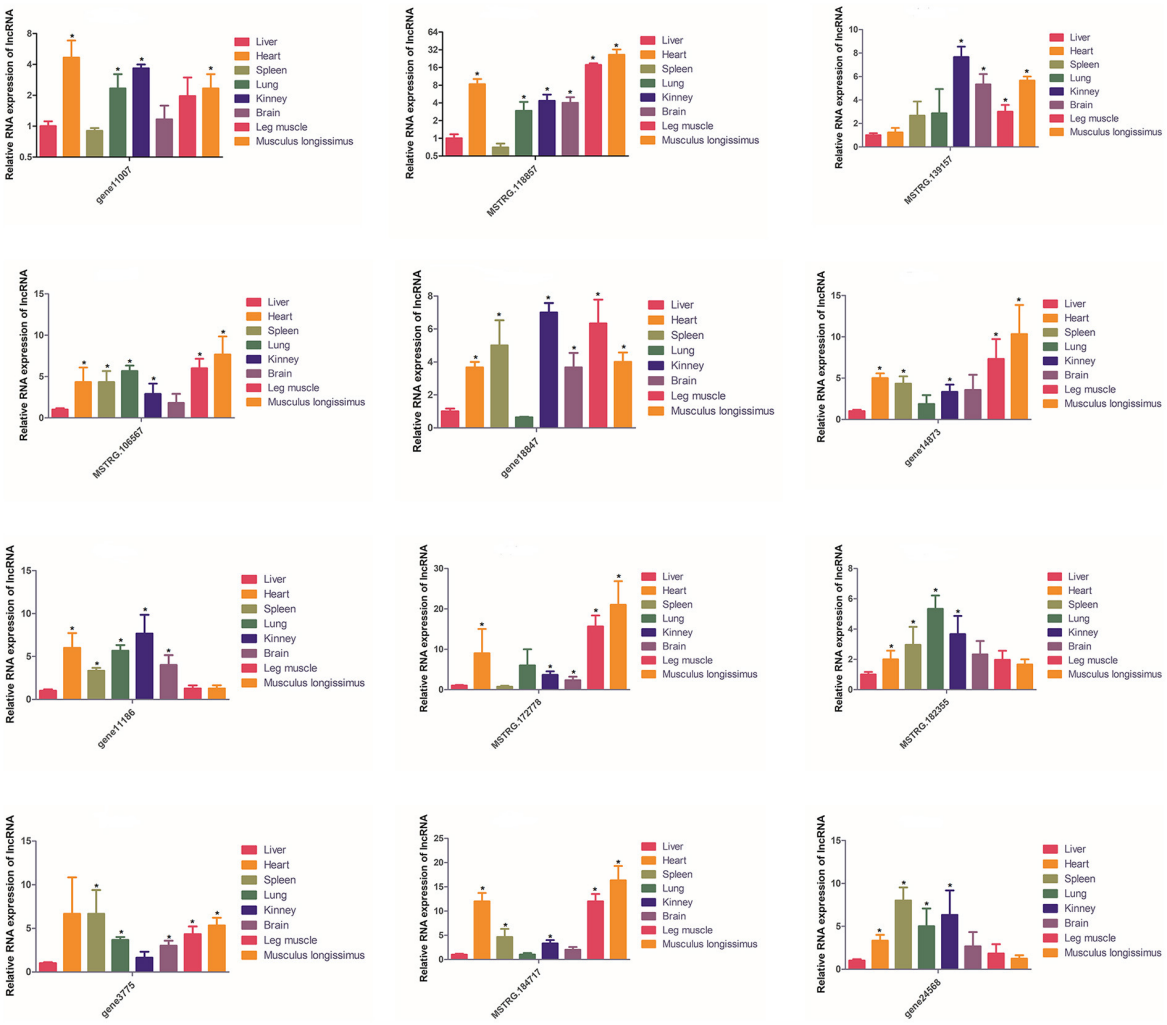
Expression levels of 12 candidate lncRNAs in different tissues obtained from adult buffaloes. The values are means ± SEMs for three individuals. *represents *P* < 0.05.

The expression of these 12 lncRNAs in the liver, brain, heart, spleen, lungs, kidneys, leg muscles and longissimus muscles of buffalo were measured during the fetal and adult stages. In fetal buffalo it was found that MSTRG.118857, gene11007, MSTRG.182355 and MSTRG.106567 were specifically expressed in skeletal muscles, and had only low expression in other tissues, indicating that these lncRNAs may play important functions in skeletal muscle development (Figure 6). Similarly, we found that MSTRG.118857, gene11007, MSTRG.172778, and MSTRG.184717 were highly expressed in adult buffalo skeletal muscle tissues (Figure 7). Of the lncRNAs selected, gene11007 and MSTRG.118857 were found to be significantly plentiful in muscle than the other lncRNAs. This result was also consistent with the sequencing results (Figure 5A). Among various tissues of fetal bovine, the expression levels of gene11007 in the longissimus dorsi and leg muscles were much higher than those in other tissues (Figure 6). However, in adult buffaloes, there was a significant decrease in the muscle content of gene11007. The content of MSTRG.118857 in muscle did not change significantly, while that of MSTRG.118857 in muscle was still significantly higher than that of other tissues (Figure 7). This means gene11007 may be specifically expressed in the fetal bovine stage of buffalo. That is, it may play a special role in the proliferation stage of buffalo muscle cells. Hence, gene11007 could be chosen as a candidate gene to explore it specific role in muscle development both *in vivo* and *in vitro*.

### Effects of gene11007 on buffalo myoblasts differentiation

**Figure 8:**
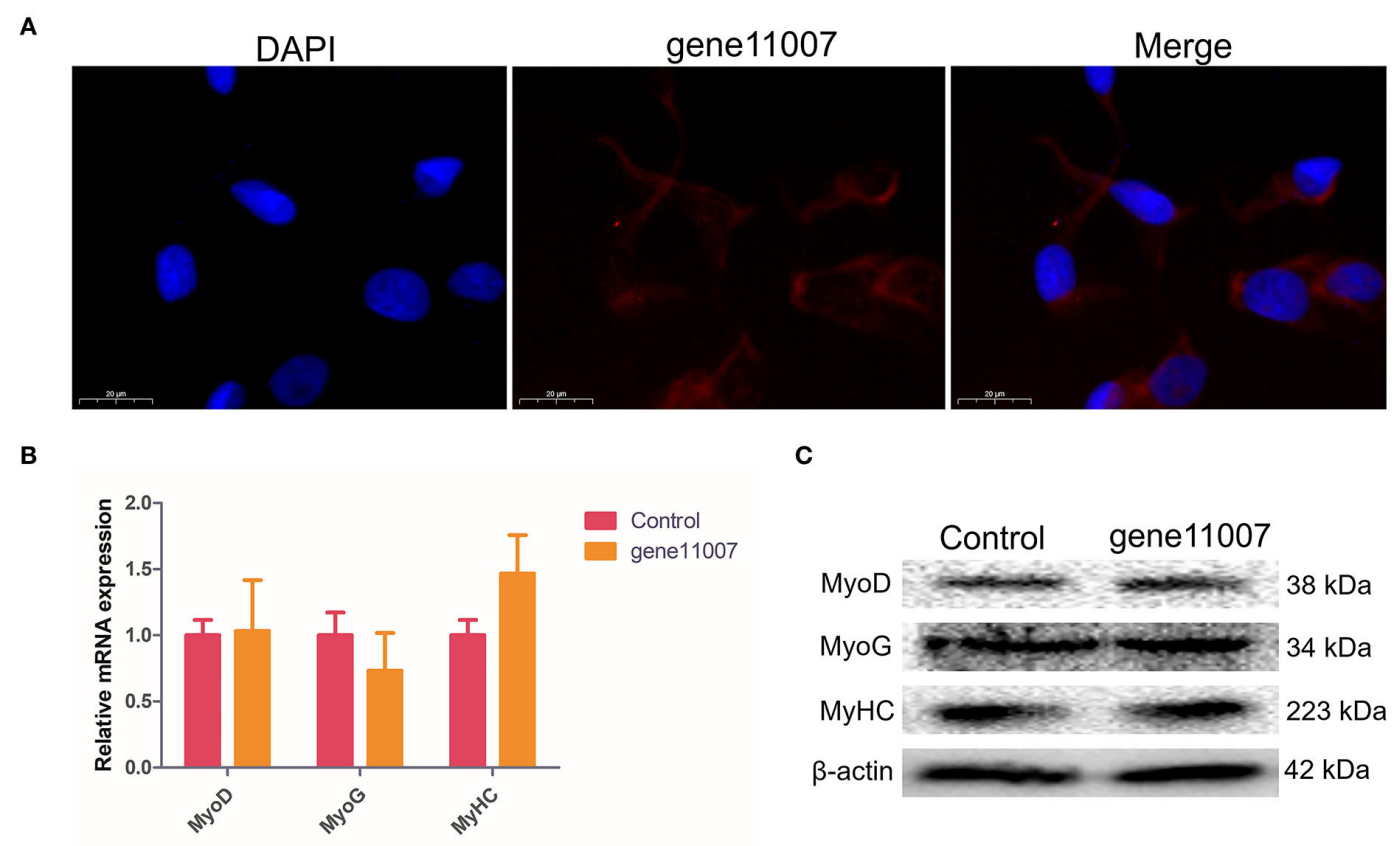
Detection of buffalo myoblasts differentiation-related proteins by overexpression of gene11007. **(A)** The results of fluorescence *in situ* hybridization showed that gene11007 was mainly localized in the cytoplasm. **(B,C)** The expression of marker genes MyoD, MyoG and MyHC for myocyte differentiation after 4 days of culture were measured by qPCR and western blotting. The values are means ± SEMs for three individuals. *represents *P* < 0.05.

FISH of gene11007 during buffalo myoblasts proliferation and differentiation showed that the gene was mainly localized in the cytoplasm (Figure 8A). In addition, a gene11007 expression plasmid was constructed and transfected into buffalo myoblasts (Supplementary Figure S1) and qPCR and western blotting techniques were used to assess the changes in expression levels of key genes, MyoD, MyoG and MyHC, during skeletal muscle development after 4 days of buffalo myoblast differentiation. The results showed that gene11007 had no significant effect on cell differentiation (*P* > 0.05; Figures 8B,C). However, the qPCR results of MyoG showed that gene11007 had a trend of promoting the differentiation of buffalo myoblasts, in contrast to MyoD and MyHC. First, the significance of the results with respect to MyoG was not obvious, indicating that this contribution was minimal. Second, MyoD acts during embryonic development and is responsible for activating myoblasts. The product of MyHC is myosin heavy chain, which is the main structure and contraction protein of skeletal muscle. MyoG regulates myogenesis by controlling the fusion of myoblasts and the formation of muscle fibers, which are key factors in the terminal differentiation of muscle cells. Therefore, the action modes of the three marker genes for muscle cell differentiation are different, and the action relationship between them is not necessary. Therefore, gene11007 may have a negative regulatory effect on MyoG, resulting in such an opposite trend.

### Effects of gene11007 on cell proliferation

**Figure 9:**
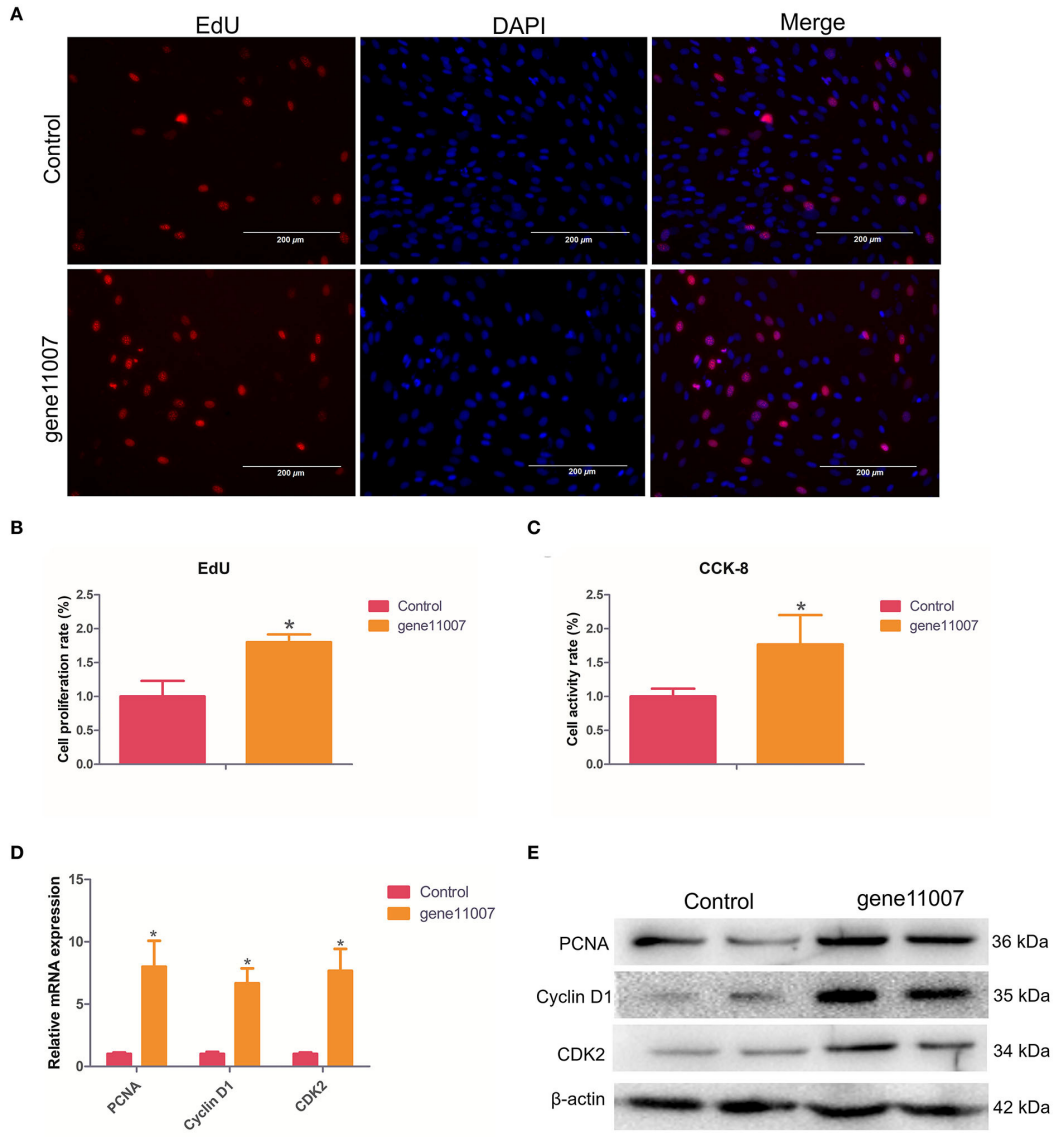
Assessment of buffalo myoblast proliferation by overexpression of gene11007. **(A,B)** EdU assays were performed with 5 visual fields for each group. **(C)** CCK-8 cell proliferation assays, *n* = 5. **(D)** Quantitative measurements of mRNA expression of key genes during cell proliferation. **(E)** Protein expression ofkey genes during cell proliferation. The values are means ± SEMs for three individuals. *represents *P* < 0.05. The scale bars represent 200 μm.

EdU staining analysis showed that compared with the control group, gene11007 increased the proportion of EdU-positive cells, indicating that this gene had a promoting effect on cell proliferation (Figures 9A,B). In addition, by using the CCK-8 assay, it was found that gene11007 could significantly induce cell viability during cell proliferation (*P* < 0.05; Figure 9C). QPCR and western blotting were used to assess the effect of key proteins, PCNA, cyclin D1 and CDK2, on cell proliferation. It was found that gene11007 increased the expression of PCNA, cyclin D1 and CDK2 at the mRNA and protein levels (Figures 9D,E). These results suggest that gene11007 promoted buffalo myoblasts proliferation.

## Discussion

The swamp buffalo is widely distributed in southern China and plays an important role in agricultural farming. In recent decades, the increased levels of mechanization used in farming, has gradually replaced the labor value of buffaloes. However, the meat from buffaloes has alien consistency and delicate quality, and is potentially a good prospect for improving through cattle cross-breeding (34–36). Skeletal muscle accounts for about 40% of the body weight of mammals. Therefore, understanding the development of skeletal muscle is of great significance for improving the best meat traits of the Chinese buffalo. However, this is not readily feasible by performing *in vivo* studies. Therefore, we used cultured myoblasts *in vitro* to analyze the effects of lncRNAs on cell differentiation. In this study, the Ribo-Zero RNA-Seq method was used to explore the lncRNA expression profiles of buffalo myoblasts during the proliferation and differentiation phases. 9,978 candidates were found in myoblasts samples with 1,576 lncRNAs that were differentially expressed between the proliferation and differentiation phases (30–33).

Studies have shown that lncRNAs regulate gene expression during the growth and development of different tissues in mammals, including muscle. LncRNAs can have a variety of regulatory mechanisms depending on its localization in the cell. In the nucleus, lncRNAs can act molecular scaffolds or decoys so as to bind regulatory elements or they can recruit transcription factors to mediate gene transcription (37–40). In the cytoplasm, lncRNAs can act as ceRNAs to competitively bind to miRNAs, thereby reducing their inhibitory effects on target gene (41–45). However, these molecular regulation mechanisms affecting muscle development are still unclear.

MSTRG.118857 and gene11007 had the highest expression levels among all up-regulated and down-regulated lncRNAs, respectively, and therefore, they may be potentially important in modulating myoblast differentiation. Tissue expression analysis of buffaloes showed that MSTRG.118857 and gene11007 were specifically expressed in skeletal muscles with relatively low expression in other tissues, indicating their potential roles in muscle development. On further study it was found that gene11007 was mainly located in the cytoplasm, indicating that it may play a role in binding to RNA-binding proteins or miRNAs. Overexpression of gene11007 in buffalo myoblasts was shown to promote cell proliferation, indicating that it plays a regulatory role in myogenesis (46).

## Conclusions

This study aimed to map the expression of mRNAs and lncRNAs in buffalo myoblasts during proliferation and differentiation. A large number of lncRNAs were identified, and several of them were highly and specifically expressed in buffalo myoblasts and skeletal tissues. Gene11007, was one of the most down-regulated lncRNAs during buffalo myoblasts differentiation. Our research provides genetic resources for in-depth understanding of the regulatory effects of lncRNAs in muscles as well as a new perspective for screening molecular targets for buffalo breeding.

## Data availability statement

The datasets presented in this study can be found in online repositories. The names of the repository/repositories and accession number(s) can be found in the article/supplementary material.

## Ethics statement

The animal study was reviewed and approved by the Institutional Animal Care and Use Committee at the College of Agriculture, Ningxia University.

## Author contributions

YM, NZ, and HL designed the experiments. SW, YZ, GX, and XW collected the experimental tissues. GX, MJ, SD, and PS analyzed the data and interpreted the results. GX and YM wrote the manuscript with input from all the authors. GX, XW, and MJ participated in designing the structure of the article. All authors read and approved the final manuscript.

## Funding

This work was supported by the Guangxi Natural Science Foundation (AD20159062 and 2020GXNSFBA297148), the National Natural Science Foundation of China (32072720, 31802043, and 31672403), Key Research and Development Projects in Ningxia Hui Autonomous Region (2019YCZX0068, 2021BEF01002, and 2021NXZD1), Autonomous Region Science and Technology Innovation Leading Talent Training Project in Ningxia (2020GKLRLX02), and the Leading Talents Fund in Science and Technology Innovation in Henan Province (No. 194200510022).

## Conflict of interest

Authors NZ and SD were employed by Guangxi Guiken Animal Husbandry Co., Ltd. The remaining authors declare that the research was conducted in the absence of any commercial or financial relationships that could be construed as a potential conflict of interest.

## Publisher's note

All claims expressed in this article are solely those of the authors and do not necessarily represent those of their affiliated organizations, or those of the publisher, the editors and the reviewers. Any product that may be evaluated in this article, or claim that may be made by its manufacturer, is not guaranteed or endorsed by the publisher.

## Supplementary material

Supplementary Table S1 The primers used for quantitative real time PCR in this study.

Supplementary Table S2 LncRNAs when identified by transcriptome sequencing.

Supplementary Table S3 MRNAs when identified by transcriptome sequencing.

Supplementary Figure S1 The efficiency of gene11007 overexpression vector.

Supplementary Text S1 The gene11007 sequence in the buffalo genome.
