# Supplementary material for: Buffalo long non-coding RNA gene11007 promotes myoblasts proliferation
Source: Front Vet Sci. 2022 Aug 5;9:857044. doi: 10.3389/fvets.2022.857044 (PMC9404873; doi:10.3389/fvets.2022.857044)
Supplement: Supplementary Table S1 — The primers used for quantitative real time PCR in this study. [file Table_1.DOCX]

**Table S1 primers for qPCR**

| **LncRNA** | **Forward primer 5’→3’** | **Reverse primer 5’→3’** |
| --- | --- | --- |
| β-actin | CTGGCATTGTCATGGACTCTG | GCTCGGCTGTGGTGGTAAA |
| gene11007 | ATTCAGCCTTGAGCACATC | GGTCCTCAGTTATAGACGAAAC |
| MSTRG.118857 | GTTGCGACTACTCATTCCTA | TACTGTCTGTTGCCTGTTCAT |
| MSTRG.139157 | CGGCATCAATATGGTGACCTC | GGCGCGATCCCACTACTG |
| MSTRG.106567 | CTGGCTGGCTAGGCGCTCCAT | TGGATGTGTCTGGAGTCTTGG |
| gene18847 | GCGTGGACAGAACTGTGGGT | CCTGCGTCTAGCCACTCTTGA |
| gene14873 | TGTTGATTCGGCTGATCTGGC | TATACCCTTGACCGAAGACCG |
| gene11186 | CTCTGTGGCGTGTTTCTCCTTCTC | GAGAGCCGAGGATGAGGAAGAGAT |
| MSTRG.172778 | TTGGCTGATATGGAACCTGACACA | CCTCTGATTGGTCTGGAGTCTTGT |
| MSTRG.182355 | TGCCATCCAGCCATCTCATCCT | GCCACCTCATGCGAAGAGTTGA |
| gene3775 | AAGTGGAGGAAGAAGCGAATG | GTAGGTCTCGCAAAGGTGGTC |
| MSTRG.184717 | GACTCGCTGAATCGCCCAGTT | GCTCCTCGGGACTCATCCAAG |
| gene24568 | ATGCTGGAACTTGTAGTGCTCAGA | GCGAATAACCCTGTGAATAGCGA |
| pcDNA-gene11007 | CCCAAGCTTTGTCCTTATTTGGCTGATATGG | GGGGTACCACAGATCGCGTAGTTGGGAGAT |
