# Supplementary figures and images for: Buffalo long non-coding RNA gene11007 promotes myoblasts proliferation
Source: Front Vet Sci. 2022 Aug 5;9:857044. doi: 10.3389/fvets.2022.857044 (PMC9404873; doi:10.3389/fvets.2022.857044)

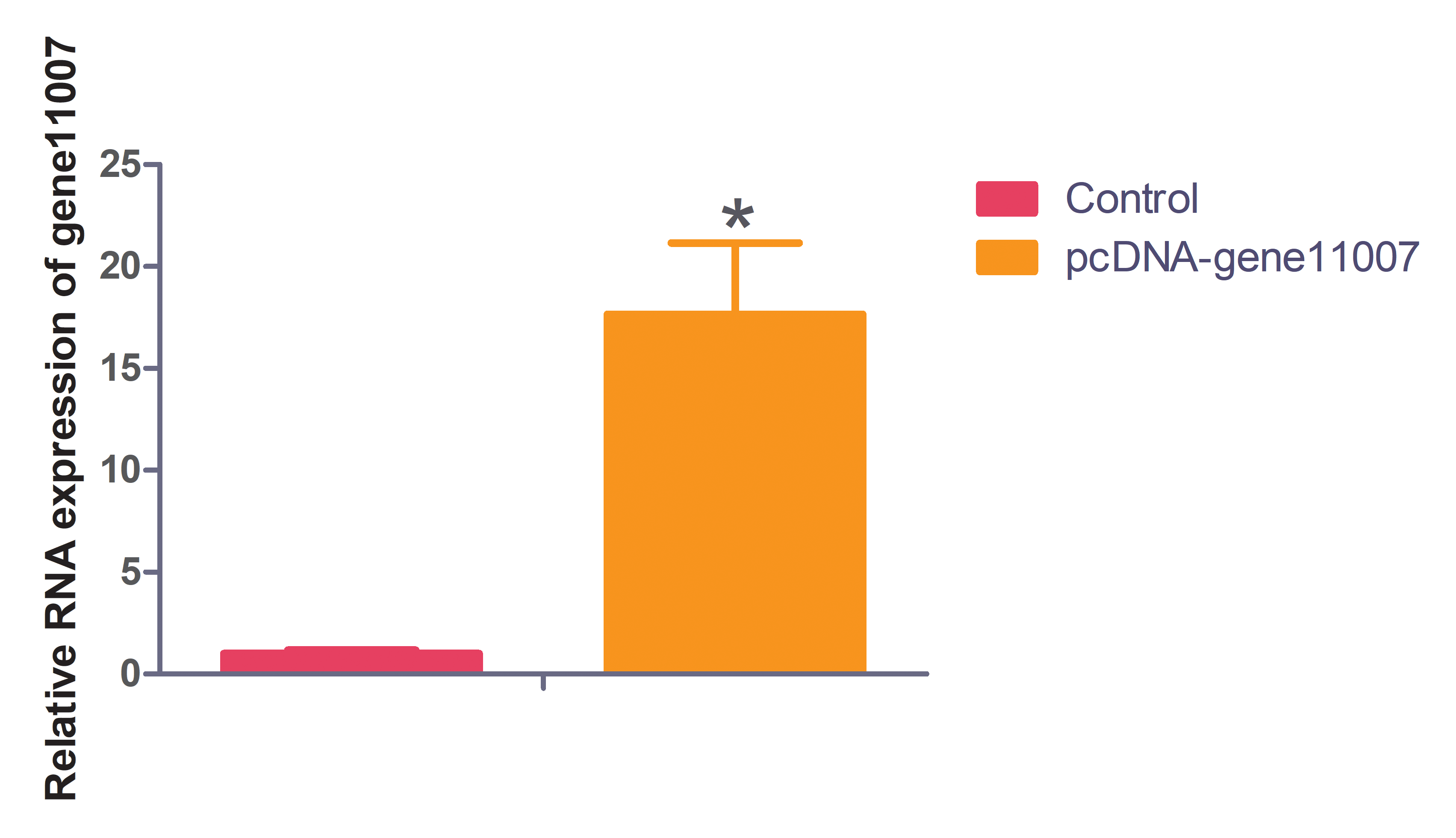

Supplement: Supplementary Figure S1 — The efficiency of gene11007 overexpression vector. [file Image_1.TIF]
