# Supplementary material for: Buffalo long non-coding RNA gene11007 promotes myoblasts proliferation
Source: Front Vet Sci. 2022 Aug 5;9:857044. doi: 10.3389/fvets.2022.857044 (PMC9404873; doi:10.3389/fvets.2022.857044)
Supplement: Supplementary Text S1 — The gene11007 sequence in the buffalo genome. [file Table_4.DOCX]

**gene11007**

TTTATGTCCTTATTTGGCTGATATGGAACCTGACACAGCTCCTGCATTCTGTCATGGGGAGTTCCGATGTATAAATATGGGGAAGCAGGCTGGAGCTGCCACTGACAGCTAACCACACAAGACTCCAGACCAATCAGAGGACGACAGTCAACAGGAAGAGAATAAGGTTGTGACAAGTCTAACCAGGATGAGAGCAGGTCAGAGGATTCCAGGGCTTCCAGCAGCTACAAAAGGGGATGGTGCATGGGAGATTACATGGCTACTGACCCACTGTCCCCACTGCCGGTGGTAGAAGCACTTCATCTCATCAACTTTTCTGTTTTCAAAGGCTTTATTCTTCCTGTATGGATGCAAGAGATGAACCATAAAGAACGCTGAGCACCAAGGAATTGATGCTTTTGAACTGTGGTGTTGGAGGAGACTCTTGAGAGTCCCTTGGACTGCAAGATCAAGCCACTCAATCCTAAAGGAAATCAGTCCTGAATATTCATTGGAAGGACTGATGCTGAAGCCGAAGCTCCAATACTTTGGCCACTTGATGTGAAGAACTGACTCACTGGAAAAGACCATGATGCTGGGAAAGTTTGAAGGCAGGAGGAGAAGGGGACAACAGAGGATGAGATGGTTGAATGGCATCACTGACTCAATGTACATTAGTTTGAGCAAGCTCCAGGAGTTGGCGATGGACAGGGAAGCCTCCCGTGCTGCAGTTCACGGGGTCACAAAGAGCTGGACACAACTGAGCAACTGAACTAACTGAACTGAGACAGACATTAATTCAGCCTTGAGCACATCAATTAGAAAATGTTTGCTCTACAAAATTATAGACCTCACTCTCCTTTTAAAAGGAATGTCACCTTTTAAAGTTTCGTCTATAACTGAGGACCAATTCATAAAGAAAGACTATAACTAGTAACTTTTTTCTCCAAAGCACATAATGCATTAAAACTTATGCAAAATTTCTTCAAAAAATAAGAATGTTTGCTATTTTAATCATCATGTTCAGCTTTAATCTCCCAACTACGCGATCTGTTTTAAACTATCTATTCTTTAAAGAAAAAAA
